# Publisher Correction: Parent of origin genetic effects on methylation in humans are common and influence complex trait variation

**DOI:** 10.1038/s41467-019-10155-7

**Published:** 2019-05-01

**Authors:** Yanni Zeng, Carmen Amador, Charley Xia, Riccardo Marioni, Duncan Sproul, Rosie M. Walker, Stewart W. Morris, Andrew Bretherick, Oriol Canela-Xandri, Thibaud S. Boutin, David W. Clark, Archie Campbell, Konrad Rawlik, Caroline Hayward, Reka Nagy, Albert Tenesa, David J. Porteous, James F. Wilson, Ian J. Deary, Kathryn L. Evans, Andrew M. McIntosh, Pau Navarro, Chris S. Haley

**Affiliations:** 1 MRC Human Genetic Unit, Institute of Genetics and Molecular Medicine, University of Edinburgh, Edinburgh, EH4 2XU UK; 2 The Roslin Institute and Royal (Dick) School of Veterinary Sciences, University of Edinburgh, Edinburgh, EH25 9RG UK; 3 Centre for Cognitive Ageing and Cognitive Epidemiology, University of Edinburgh, Edinburgh, EH8 9JZ UK; 4 Centre for Genomic and Experimental Medicine, IGMM, University of Edinburgh, Edinburgh, EH4 2XU UK; 5 Edinburgh Cancer Research Centre, Institute of Genetics and Molecular Medicine, University of Edinburgh, Edinburgh, EH4 2XR UK; 6 Centre for Global Health Research, Usher Institute of Population Health Sciences and Informatics, University of Edinburgh, Edinburgh, EH8 9AG UK; 7 Department of Psychology, University of Edinburgh, Edinburgh, EH8 9JZ UK; 8 Division of Psychiatry, University of Edinburgh, Edinburgh, EH10 5HF UK

**Keywords:** DNA methylation, Epigenomics, Genome-wide association studies, Quantitative trait

Correction to: *Nature Communications* 10.1038/s41467-019-09301-y, published online 27 March 2019.

In the original version of this Article, the legend in the upper panel of Fig. 2 incorrectly read ‘paternal imprinting’ and should have read ‘maternal imprinting’. This has been corrected in both the PDF and HTML versions of the Article.

