# Supplementary material for: Parent of origin genetic effects on methylation in humans are common and influence complex trait variation
Source: Nat Commun. 2019 Mar 27;10:1383. doi: 10.1038/s41467-019-09301-y (PMC6437195; doi:10.1038/s41467-019-09301-y)
Supplement: Supplementary file 1 — Supplementary Information [file 41467_2019_9301_MOESM1_ESM.pdf]

Supplementary Methods, Supplementary Figures and Supplementary Tables for the manuscript entitled ‘Parent of origin genetic effects on methylation in humans are common and influence phenotypic variation’ by Zeng *et al.*, 2019

Supplementary Method 1. Quality control, phasing and imputation in GS:SFHS dataset.

Quality control (QC) of genotyped SNPs used the following inclusion thresholds: missing SNPs per individual ≤2%, SNP genotype call rate ≥98%, minor allele frequency (MAF) >5% and Hardy-Weinberg equilibrium p-value > 1 x 10^{-6}. Before imputation, haplotypes were inferred with SHAPEIT with the option --duohmm which refines the phasing by taking into account pedigree information [1]. Imputation was performed following the Sanger Imputation Service pipeline (https://imputation.sanger.ac.uk/) using the Haplotype Reference Consortium (HRC) reference panel release 1.1[2]. Only imputed SNPs with info score ≥ 0.8 and MAF ≥ 0.01 were used in downstream analysis.

Supplementary Method 2.Quality control, normalization and pre-correction for DNA methylation data in GS:SFHS

Quality control (QC) of the DNA methylation was carried out before normalisation. The R package shinyMethyl was used to perform preliminary QC [3]. The filtering process removed 81 samples, including 1) outliers based on overall array signal intensity and control probe performance, 2) samples showing mismatch between recorded gender and predicted gender based on X and Y chromosome DNA methylation, and 3) genetic ethnic outliers identified by a principal component analysis for the GS:SFHS cohorts [4]. An additional QC was performed using the pfilter function in the R package watermelon [5] based on the following criteria: samples are removed if ≥ 1% sites have a detection p-value of > 0.05. Probes are removed from the dataset if: 1) they have more than 5 samples with a bead count <3; or 2) if ≥ 1% of the samples have a detection p-value of > 0.05; or 3) they contain a SNP of MAF >1% in the European population in the 1000 Genome Project [6]; or 4) they are predicted to cross-hybridise [6, 7]. This removed 18 further samples and 88,728 probes, leaving 5,101 samples and 778,108 probes to be used in the normalization step. Before normalization, individual probe-sample pairs with a detection p-value of > 0.05 were removed. Normalization was performed using function preprocessNoob in the R package minfi [8]. This produced data of two formats: 1) Beta values which measure the ratio between methylated intensity and sum of methylated and unmethylated intensity, ranging from 0 to 1 and measuring the percentage of methylation and 2) M-values which are the logit transformation of Beta values. M-values were used in downstream analysis as a previous study suggested was more appropriate[9].

In order to remove potential effects from technical factors, we used linear mixed modelling to pre-correct each QCed probe for fixed and random effects (fixed effects: top 50 principal components of control probe intensities (explaining 99% of variation in control probe intensities) [10], appointment clinic centre, processing batch, year of the visit, sentrix position (position of the sample in Illumina slide), the appointment

day of the week (ranges from 1 to 7); random effects: the appointment date and the sentrix ID (Illumina slide). The model converged successfully for 639,238 sites, and the produced Residualised-M-values were used as DNA methylation phenotypes in downstream analysis. For individual sites, outlier samples with Residualised-M-values more than five interquartile ranges from the nearest quartile were removed [11]. Cell count was estimated for granulocytes, monocytes, B-lymphocytes, natural killer cells, CD4+ T-lymphocytes and CD8+ T-lymphocytes using the estimateCellCounts() function in R package minfi[8], and was corrected in later analysis stages (described in the main text).

Supplementary Method 3. Identify parent of origin information for alleles transmitted to the offspring

In GS:SFHS, haplotypes were extracted from phased imputation files in vcf format (phasing and imputation are described above). Among participants with genotype data, there were 2,680 trios (i.e. both parents and one offspring), 1,185 father-offspring duos, and 3,274 mother-offspring duos, from which we inferred parent-of-origin information for alleles in the offspring by comparing offspring haplotypes to their parents' using informative loci (heterozygous in offspring). Trios were used to establish the threshold for duos. We first estimated the pairwise haplotype similarity between haplotypes in parents and offspring in trios, through which the similarity threshold for assigning transmitted and non-transmitted haplotypes was established. We then applied the same threshold as estimated in trios to assign transmitted haplotypes in duos. Offspring (N=34) for which haplotype similarity with parents is more than two interquartile ranges from the nearest quartile were removed. This resulted in 7,106 offspring with parent-of-origin information successfully assigned to haplotypes and hence alleles at single loci. We evaluated the accuracy of the assigned parent-of-origin haplotype at a genotype level by checking consistency in each informative SNP in each duo/trio (duos: heterozygous SNP in offspring must be homozygous in the genotyped parent, trios: heterozygous SNP in offspring must be homozygous in at least one parent). We found an accuracy of over 99.9% across all SNPs.

Supplementary Method 4. Clumping for methylation sites

Methylation sites were ranked based on their p-values for POE in the selected model in variance component analysis described above. Starting with the site with the most significant POE ('index site'), we computed the correlation of methylation values (adjusted for technical variables and age, age^2, gender, cell count, season of the visit, appointment time of the day, appointment day of the week) between this site and other sites within 250 kb upstream and downstream of this 'index' site. Any non-index site within the window having an $R^2 > 0.1$ with the index site was assigned to the same clump as the index site. The procedure is repeated with the next most significant site not assigned to previous clumps. Each site can only appear in a single clump.

Supplementary Method 5. Designed variance-covariance matrices representing different sources of variation in DNA methylation

<u>G matrix:</u> Genomic relationship matrix that was created in GCTA[12]. Genotypes from N common SNPs (in this case, all common SNPs that passed quality control) were used to estimate the genetic relationships between individuals j and k using the formula below:

$$A_{jk} = \frac{1}{N}\sum_{i=1}^{N}\frac{(x_{ij}-2p_i)(x_{ik}-2p_i)}{2p_i(1-p_i)}$$

where x was the dosage of the minor allele for individual j or k at SNP i. p_i was the reference allele frequency of SNP i.

<u>K matrix</u>*:** Kinship relationship matrix obtained through modification of the ***G matrix. Entries with relationship coefficients less than or equal to 0.05 (representing distantly related individuals) in ***G*** matrix were set to 0 in ***K*** [13].

***<u>Family Environmental similarity matrices</u>*:** represented shared environmental effects or non-additive genetic effect for different familial relationships:

F matrix**:** similarity matrix describing nuclear families (individuals having lived or living in the same household)
S matrix: environmental similarity matrix representing full-siblings relationships (may represent shared environmental effect or non-additive genetic effect between full siblings)
C matrix: environmental similarity matrix representing couple relationships.

Each familial environment similarity matrix was a N x N matrix where diagonal entries were set to 1, off-diagonal entries for two individuals sharing the family environment of interest were set to 1 and the remaining off-diagonal entries were set to 0 [14].

Supplementary Method 6. Modified variance component analysis detects candidate methylation sites with parent-of-origin inheritance pattern.

Rationale behind this method:

In this study, using the GKFSC model [14], we found that the methylation sites with a genome-wide significant e_S^2 were highly enriched in genomic regions previously reported as displaying genomic imprinting ($P_{(\text{Fisher exact test})} = 1.3\text{x}10^{-80}$ when giving the known imprinted region a 2kb extension and $P_{(\text{Fisher exact test})} = 1.2\text{x}10^{-32}$ when there is no extension to the known imprinted regions. Known imprinted regions were collected from supplementary Table 2 in [15] and the newly identified regions (including both the CpGs and POE-mQTL

SNPs) reported in the same paper). A likely reason was that the inheritance of methylation in these sites follows a parent-of-origin-dependent pattern, which reduces the phenotypic covariance between parents and their offspring relative to that between full-siblings [16]. Therefore, the full-sibling associated variance component, when fitted simultaneously with additive genetic components, captures the increased similarity between full-siblings caused by parent-of-origin effects (POE), and can be used to identify methylation sites potentially influenced by POE.

However, the full-sibling relationship matrix may not fully capture the similarity patterns created by POE, given the fact that there are multiple possible imprinting inheritance patterns [17], each of which is expected to display a different covariance structure between parents and offspring and between full-siblings. Specifically, previously reported imprinting POE include two broad categories: 1) Classical paternal- or maternal- imprinting patterns and 2) complex imprinting which includes polar dominance and bipolar patterns (Figure 1) [17]. Maternal imprinting results in the effect of the maternal allele being suppressed and hence only the paternal gamete is effective which increases the covariance between fathers and their offspring and between full-siblings, but not between mothers and their offspring. Similarly, paternal imprinting increases the covariance between mothers and their offspring and between full-siblings, but not between fathers and their offspring. Complex imprinting patterns increase the covariance between full-siblings, but not between the parents and their offspring (Figure 1).

Therefore, three relationship matrices were designed to represent three imprinting patterns (Figure 1):

1) A maternal-imprinting matrix ($\boldsymbol{S_M}$) representing increased between-father-offspring and between full-sibling similarities introduced by maternal imprinting. In the matrix off-diagonal elements corresponding to the coefficients of father-offspring pairs and full-sibling pairs were set to 0.5, and the remaining off-diagonal elements were set to 0; diagonal elements were set to 1.

2) A paternal-imprinting matrix ($\boldsymbol{S_P}$) representing increased between-mother-offspring and between-full-sibling similarities introduced by paternal imprinting. In the matrix the off-diagonal elements corresponding to the coefficients of mother-offspring pairs and full-sibling pairs were set to 0.5, and the remaining off-diagonal elements were set to 0; diagonal elements were set to 1;

3) Previously defined $\boldsymbol{S}$ matrix (that was fitted in the GKFSC model, see Text s3) representing increased between-full-sibling similarities introduced by complex imprinting POE.

For each methylation site, we fit the following three linear mixed models:

（1） $\boldsymbol{Y = Xb + g_g + g_{kin} + P_s + \varepsilon}$ ***(alt1)***

(2) $\boldsymbol{Y = Xb + g_g + g_{kin} + P_{sp} + \varepsilon}$ ***(alt2)***

(3) $\boldsymbol{Y = Xb + g_g + g_{kin} + P_{sm} + \varepsilon}$ ***(alt3)***

Where $\boldsymbol{Y}$ was a vector of M-values (phenotypes with technical covariates were pre-corrected), $\boldsymbol{b}$ was a vector of biological covariates fitted as fixed effects (i.e., age, age^2, gender, cell count, season of the visit, appointment time of the day, appointment day of the week). $\boldsymbol{g_g}$ and $\boldsymbol{g_{kin}}$ were the random additive genetic effects from the SNPs and the additional random additive genetic effect from the pedigree, respectively, with $\boldsymbol{g_g} \sim N\,(0, \boldsymbol{G\sigma^2_g})$ and $\boldsymbol{g_{kin}} \sim N\,(0, \boldsymbol{K\sigma^2_{kin}})$. $\boldsymbol{P_s}$, $\boldsymbol{P_{sp}}$, and $\boldsymbol{P_{sm}}$ represented the random non additive POE introduced by complex imprinting, paternal imprinting and maternal imprinting effects, with $\boldsymbol{P_s} \sim N\,(0, \boldsymbol{S\sigma^2_S})$, $\boldsymbol{P_{sf}} \sim N\,(0, \boldsymbol{S_f\,\sigma^2_{Sf}})$, and $\boldsymbol{P_{sm}} \sim N\,(0, \boldsymbol{S_m\,\sigma^2_{Sm}})$, respectively. For each methylation site, we compared each of the three alternative models with a null model that only includes additive genetic components:

$\boldsymbol{Y = Xb + g_g + g_{kin} + \varepsilon}$ ***(null)***

and selected the best alternative model with the largest significant improvement of model fit, based on a log-likelihood-ratio test (LRT). The genome-wide analysis was performed using the default constrained mode in GREML to constrain the estimate of proportion of variance explained by each component to be positive. After analysis of each CpG site (genome-wide analysis), we retested the LRT using the unconstrained mode for CpGs that reached genome-wide significance for the tested component in GREML and only retained those CpGs that remained genome-wide significant both with the constrained and the unconstrained modes as candidates for POE-influenced CpGs. These analyses were performed in GCTA [12].

Supplementary Method 7. Replication sample for POE-PheWAS findings: UK-Biobank

The UK-Biobank recruited 501,726 individuals at 22 centres across the UK, 488,380 individuals of which have genotypic data available[18]. Imputation was performed using the HRC reference panels by IMPUTE4[18]. Quality control process included the following: SNP genotype call rate ≥95%, minor allele frequency (MAF) >5%, Hardy-Weinberg equilibrium p-value > 5 x 10^{-6} and imputation quality score (INFO) ≥ 0.8. Phenotyping for participants was performed using health records, biological sampling, physical measurement, touchscreen questionnaire and a brief verbal interview. UKB provided a list of pairs of individuals related up to the third degree as well as the estimate of pairwise kinship coefficient and the fraction of markers for which the pair shares zero alleles (IBS0). This information had been obtained using the software 'KING'[19]. Pairs whose kinship coefficient was larger than 0.175 and smaller than 0.4, IBS0 smaller than 0.001 and age difference larger than 14 were identified as parent-offspring pairs. In total, 5,197 offspring from 1,066 trios and 4,131 duos were identified. The '--duohmm' function in SHAPEIT[1] was used to identify haplotypes in a 5MB extended (both sides) region of the POE-PheWAS significant SNP. Parent-of-origin was assigned to alleles in the target SNP using the same method as in GS:SFHS, with 159 offspring removed from

the analysis as their haplotype similarity with a parent was smaller than the minimum value of that observed in GS:SFHS. After further removing non-white-British and randomly retaining one of each related offspring sets (relationships where the kinship coefficient was larger than 0.05), 4,378 offspring were used in the replication analysis for the POE-PheWAS.

Supplementary Method 8. Databases used in functional region based enrichment/depletion tests

The following databases were used in annotation:

ENCODE Chromatin genome-segmentation data for K562 and GM12878 cell lines
http://hgdownload.cse.ucsc.edu/goldenPath/hg19/encodeDCC/wgEncodeAwgSegmentation/

ENCODE Histone modification (BroadHistone) data for K562 and GM12878 cell lines
http://hgdownload.cse.ucsc.edu/goldenPath/hg19/database/

ENCODE transcription factor binding sites data (uniform set) for K562 and GM12878 cell lines
http://hgdownload.cse.ucsc.edu/goldenPath/hg19/database/

Published GWAS associations (downloaded on 19-05-2017)
http://hgdownload.cse.ucsc.edu/goldenPath/hg19/database/gwasCatalog.txt.gz

Published EWAS associations (collected from literature, see Table s5)
Substructure of genic regions (refGene database)

Supplementary Figures

Supplementary Figure 1. Patterns of potential genetic effects of one or two regulatory SNP on a methylation site. The alleles at the SNPs are A and C and G and T respectively, with genotypes indicated at the bottom of each panel (I-IV) I. Additive effect; II. Maternal POE; III. Paternal POE; IV. Complex (bipolar) POE. The cross indicates the effect of the allele is repressed by genomic imprinting. Blue: Paternal gamete; Orange: Maternal gamete. I-III: The diagrams show the effect a SNP with two alleles (A or C) that regulates the methylation level of the CpG site. Allele A leads to a higher level of methylation. Pattern I represents an additive model and patterns II and III represent maternal and paternal imprinting, respectively. We use the standard definition of maternal imprinting being where the maternal allele is silenced and the paternal expressed and vice-versa for paternal imprinting. In patterns II and III imprinting causes differential phenotypic expression in the heterozygotes. Pattern IV represents one potential complex imprinting model: a second SNP (with variants G/T) in complete linkage disequilibrium with the first SNP (A is always inherited with G, C is always inherited with T) also regulates the methylation level of the nearby CpG but shows a different imprinting pattern. Under the combined effect of the two SNPs, the methylation level of the CpG displays a bipolar imprinting pattern.

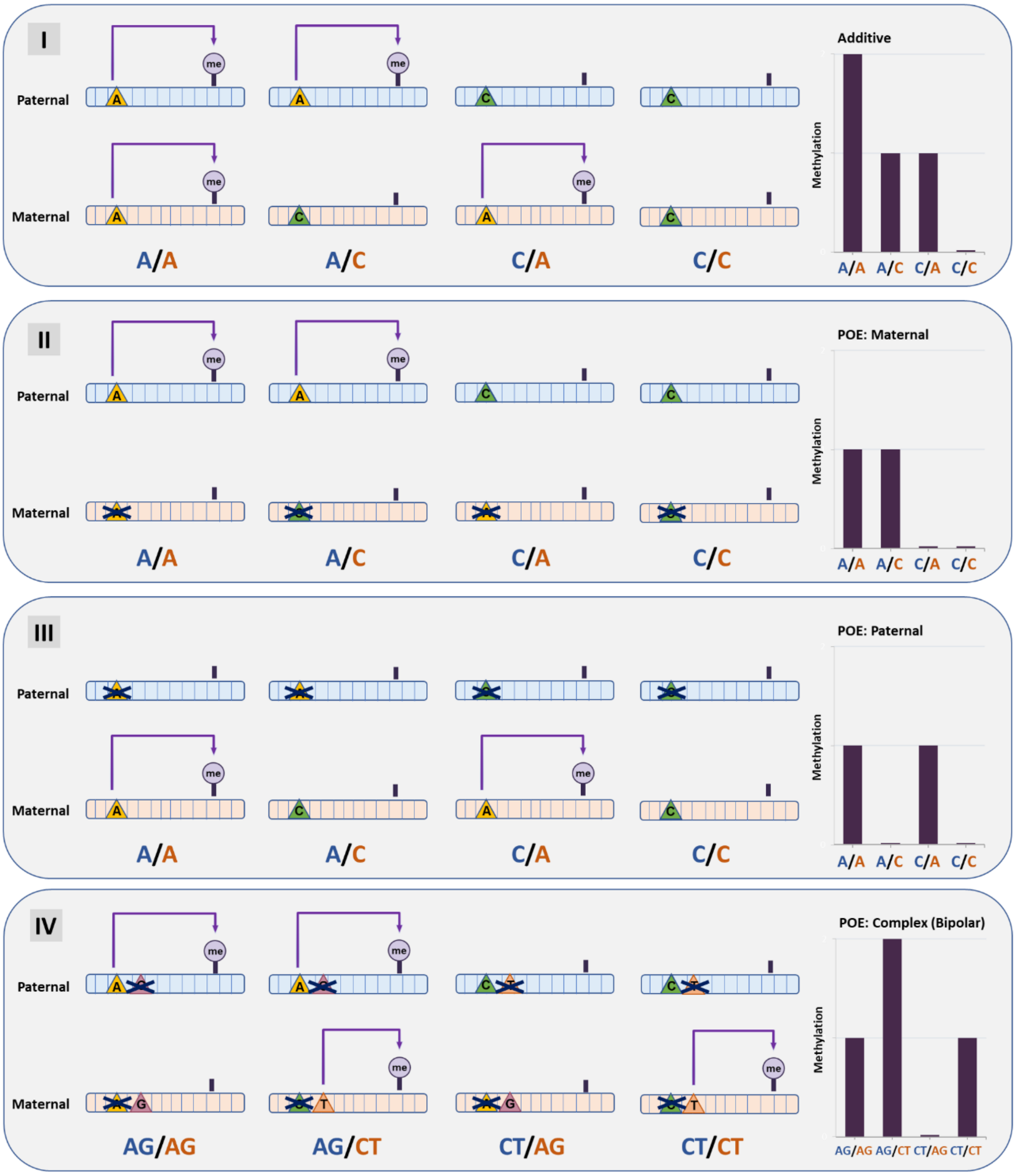

Supplementary Figure 2. Distribution of SNP heritability across all assayed CpGs located in transcription start sites and within 10 kb either side of TSSs. Boxplots: red diamond: mean; centre line: median; upper/lower hinges: the first/third quartiles; upper/lower whiskers: 1.5 inter-quartile range from the hinges.

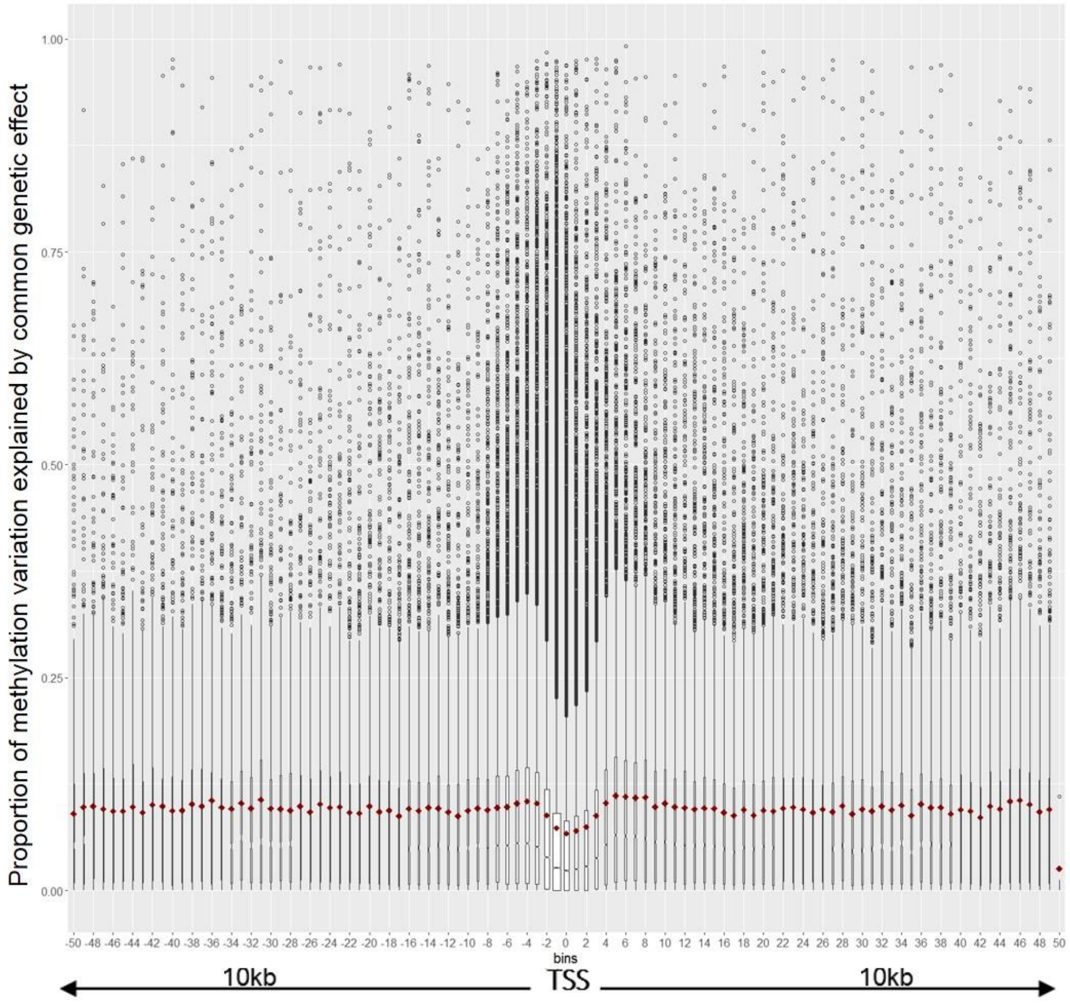

Supplementary Figure 3. Distribution of point estimates that reach nominal significance (P ≤ 0.05) for each genetic or environmental variance component as a proportion of total variance in the analysis of all CpGs.

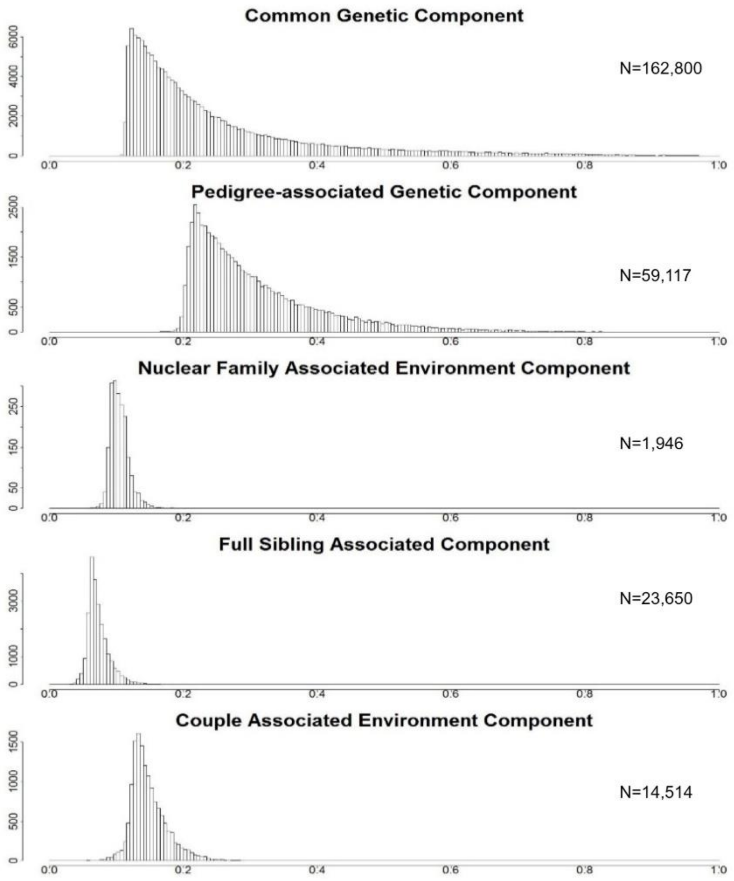

Supplementary Figure 4. A Circos* plot providing an overall view of the 984 candidate POE-influenced CpGs identified through genome-wide POE scan and their POE-mQTLs identified through POE-mQTL analysis. From centre: 1) Inner circle shows POE-mQTL analysis results, each link is a POE-mQTL-SNP-methylation-site pair, the colour of the link is the colour for the chromosome of the POE-mQTL-SNP. 2) Histograms showing modified variance component analysis results. Height: -log(Pvalue) of the POE in for 606 complex imprinting (orange), 158 paternally driven imprinting (blue), 220 maternally driven imprinting (brown) POE-influenced CpGs. 3) Outer circle: Gene names for genes overlapping with 984 POE-influenced CpGs. Red: Genes with novel evidence of POEs. *Krzywinski, M. et al. Circos: an Information Aesthetic for Comparative Genomics. Genome Res (2009) 19:1639-1645.

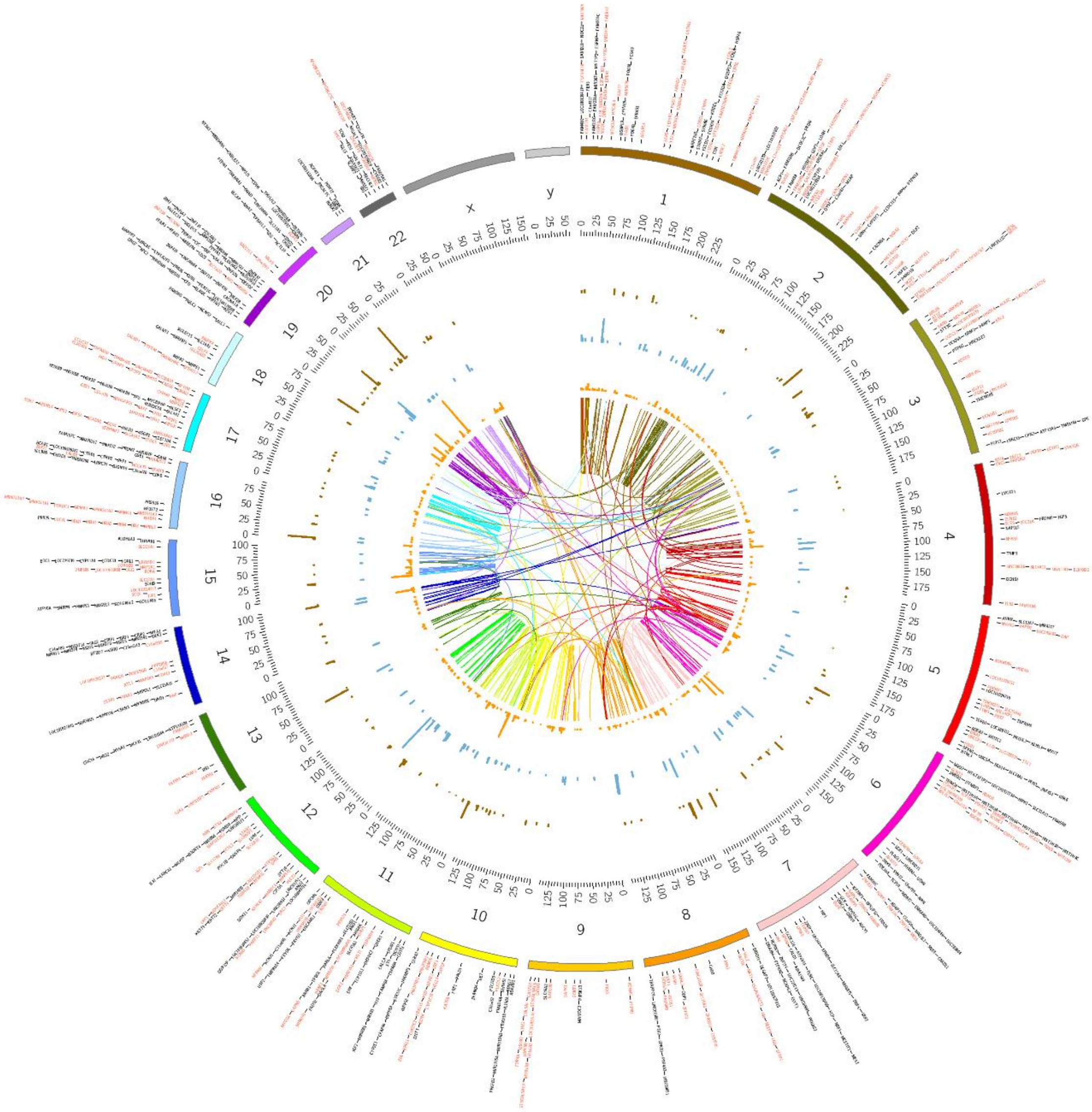

Supplementary Figure 5. (a) a POE-influenced CpG located within IGF2. (b) a POE-influenced CpG located within PEG3. A: regional plot of –log transformed P value of the POE in the selected models from the variance component analysis for CpGs in the region 20kb either side of selected CpG (the latter shown by a red-ringed black dot). The heat map shows the Pearson correlation between CpGs in this 40kb region. B: Methylation (measured by M values, corrected for technical and biological covariates) correlation between pairwise nuclear family members and p value of test of r=0. C: POE regulation through POE-mQTL for the target CpG. (NB cg21917949 in s4 (a) is regulated by two independent SNPs). Boxplots: centre line, median; box limits, upper and lower quartiles; whiskers, 1.5x interquartile range; points, outliers.

(a)

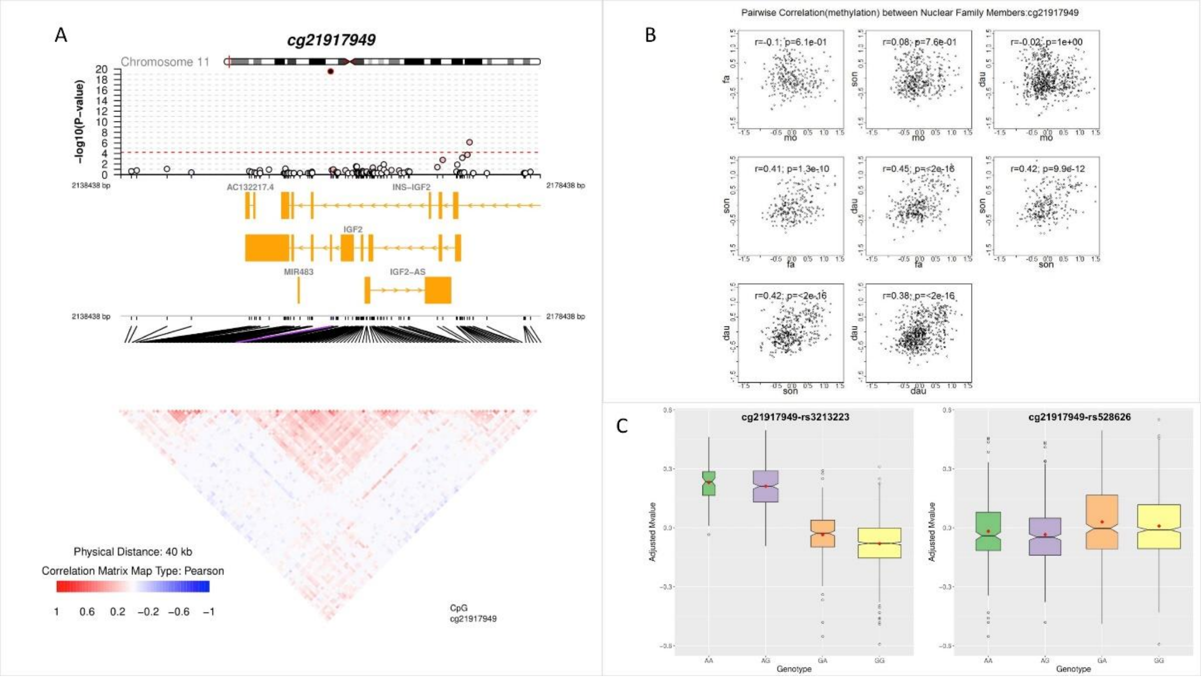

(b)

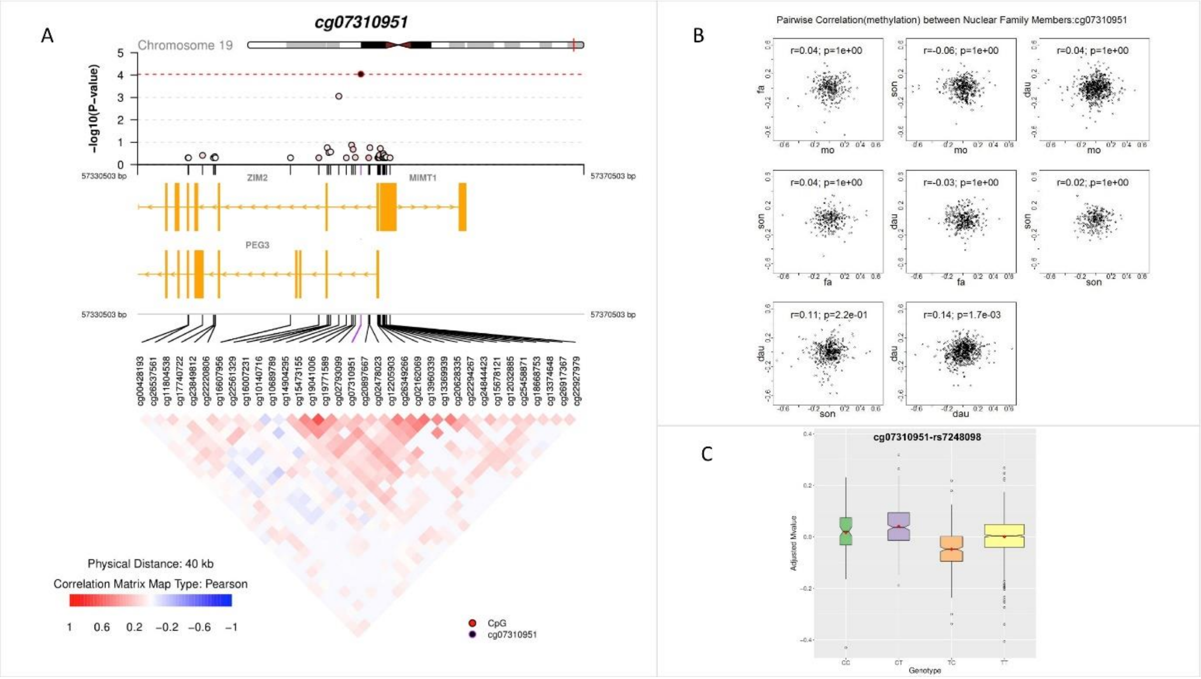

Supplementary Figure 6. a: Distribution of 984 POE-influenced CpGs in genic regions. b: Enrichment/depletion results for chromatin states in two cell lines. Error bars: 95% confidence interval.

a

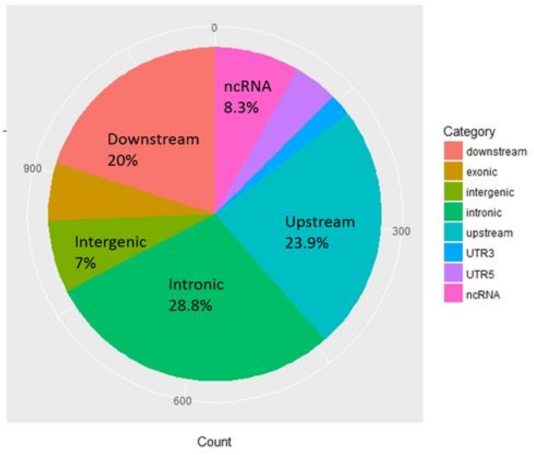

b

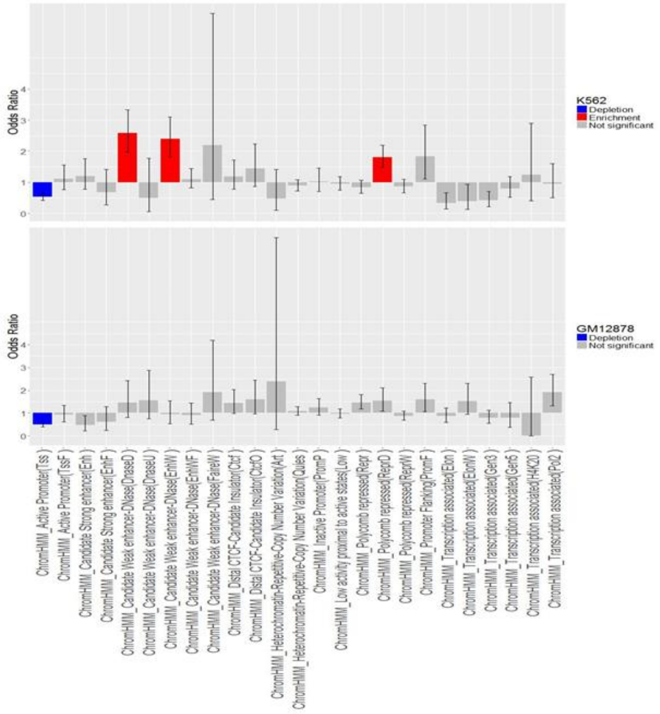

Supplementary Figure 7. Significant annotations in enrichment (red) or depletion (blue) for methylation sites. Left: CpGs with strong POE evidence; right: CpGs with moderate POE evidence. Error bars: 95% confidence interval.

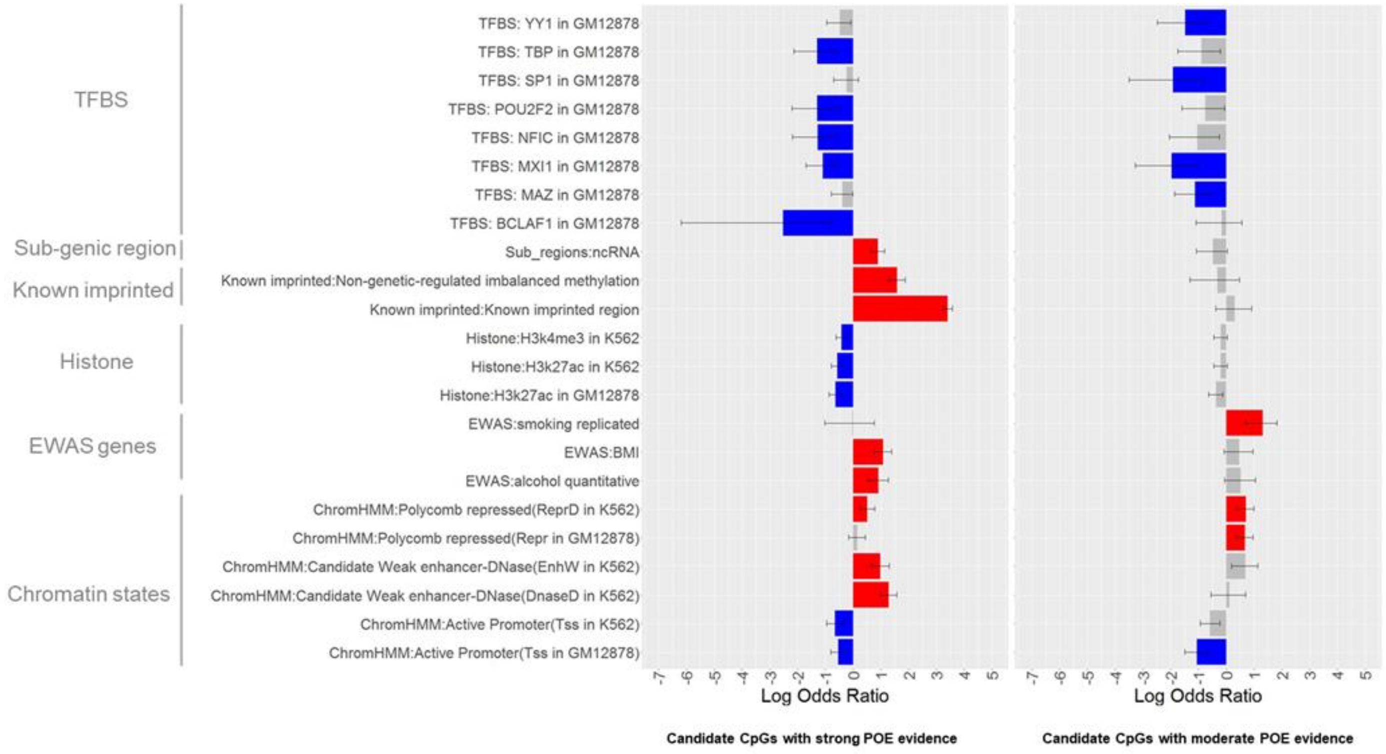

Supplementary Figure 8. QQplot for POE-PheWAS.

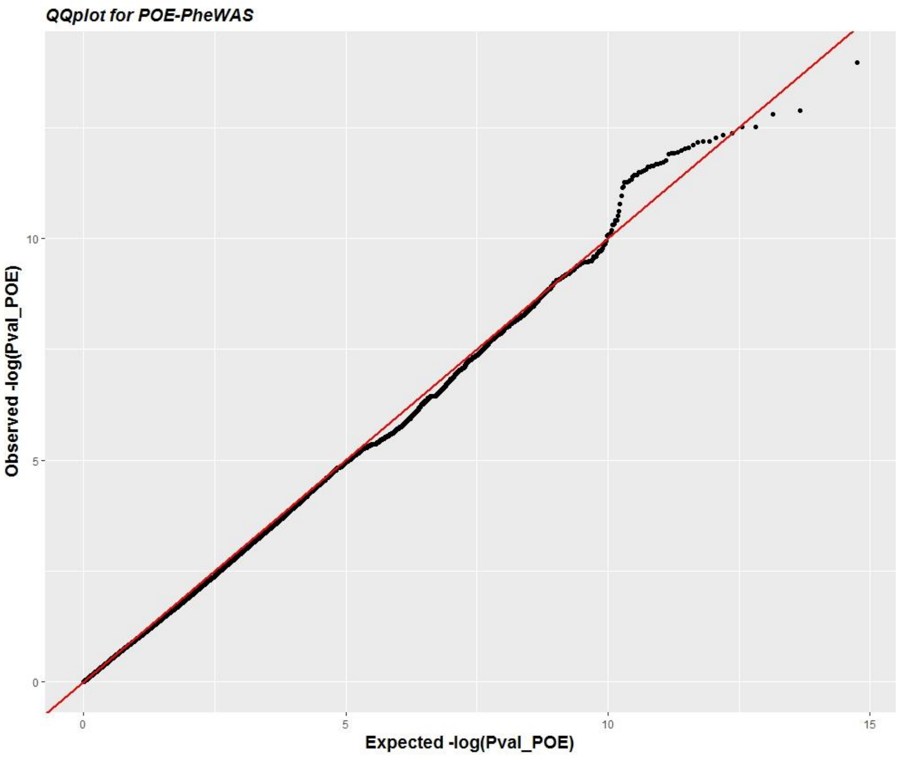

Supplementary Figure 9. POE from rs6100212 on waist circumference in different age and sex groups in GS:SFHS. Boxplots: centre line, median; box limits, upper and lower quartiles; whiskers, 1.5x interquartile range; points, outliers.

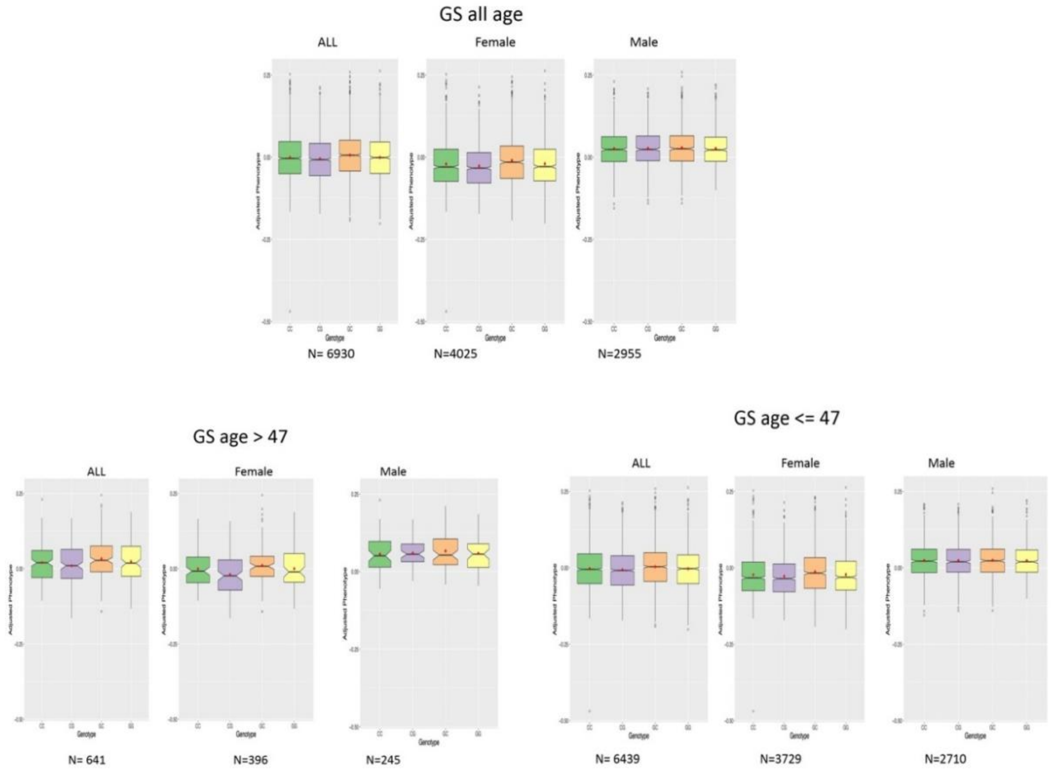

Supplementary Figure 10. Age distribution of GS:SFHS and UK-Biobank samples (offspring with parent-of-origin information assigned to SNPs) used in this study.

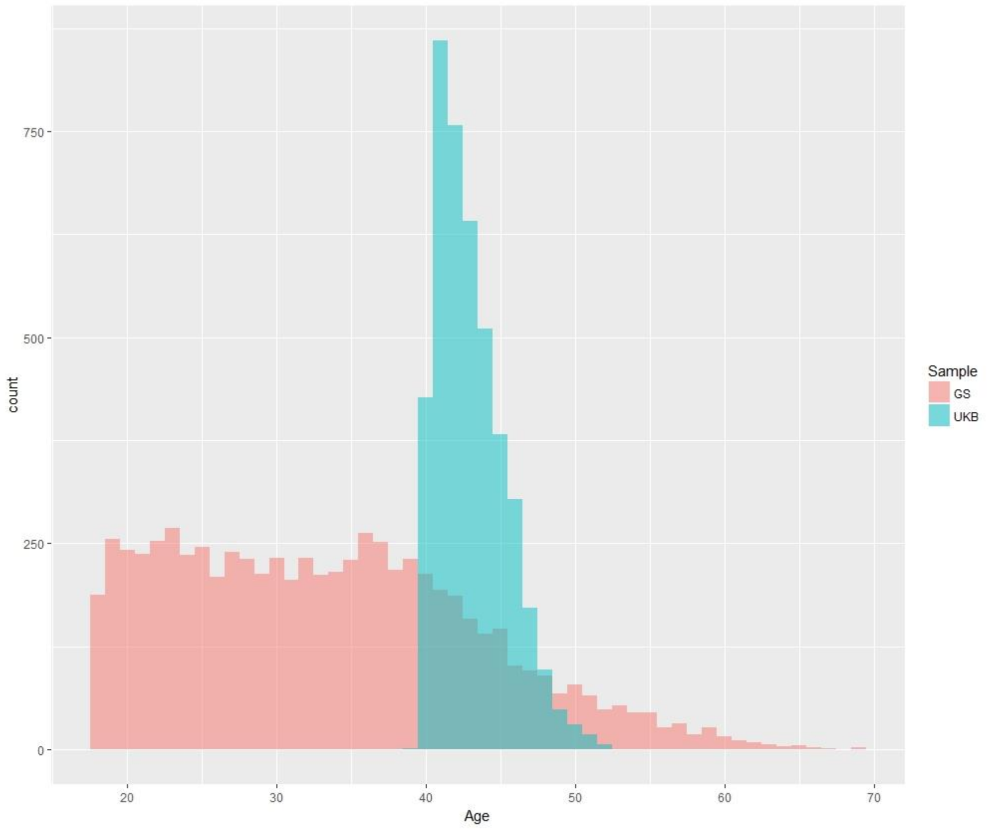

Supplementary Figure 11. An example of multiple imprinting patterns displayed in a single methylation CpG. Boxplots: centre line, median; box limits, upper and lower quartiles; whiskers, 1.5x interquartile range; points, outliers.

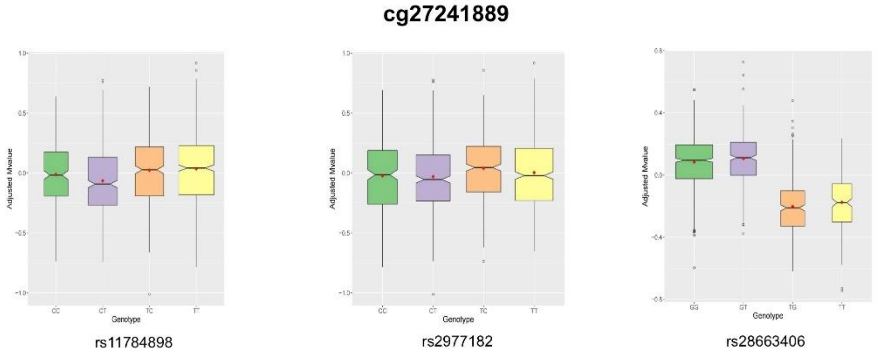

Supplementary Tables

Enrichment test for 984 candidate POE-influenced CpGs in known imprinted regions							
**Extension of known imprinted regions**	**0**	**Within 2 kb**	**Within 5 kb**	**Within 10 kb**	**Within 20 kb**	**Within 250 kb**	**Within 2 Mb**
**P (Fisher exact)**	1.60E-73	5.30E-171	2.80E-167	2.10E-159	2.60E-150	5.80E-71	4.50E-25
**Odds Ratio**	9.7 (8.0-11.7)	15.3 (13.1-17.7)	14.1 (12.1-16.3)	12.7 (10.9-14.7)	10.9 (9.4-12.6)	3.8 (3.3-4.3)	1.9 (1.7-2.1)
**N in known imprinted**	125	234	240	243	252	336	596
**N not in known imprinted**	859	750	744	741	732	654	388
**Enrichment test for 586 candidate POE-influenced CpGs (Strong POE Evidence) in known imprinted regions**							
**Extension of known imprinted regions**	**0**	**Within 2 kb**	**Within 5 kb**	**Within 10 kb**	**Within 20 kb**	**Within 250 kb**	**Within 2 Mb**
**P (Fisher exact)**	1.40E-90	3.20E-214	1.60E-209	1.00E-202	4.00E-191	1.20E-94	1.00E-29
**Odds Ratio**	16.5 (13.3-20.2)	27.6 (23.2-32.7)	27.5 (23.2-32.7)	25.0 (21.1-29.6)	21.3 (17.9-25.2)	6.4 (5.4-7.6)	2.6 (2.2-3.1)
**N in known imprinted**	116	223	227	230	235	271	395
**N not in known imprinted**	470	363	359	356	351	315	191
**Enrichment test for 398 candidate POE-influenced CpGs (Moderate POE Evidence) in known imprinted regions**							
**Extension of known imprinted regions**	**0**	**Within 2 kb**	**Within 5 kb**	**Within 10 kb**	**Within 20 kb**	**Within 250 kb**	**Within 2 Mb**
**P (Fisher exact)**	0.2	0.3	0.2	0.3	0.2	0.07	0.01
**Odds Ratio**	1.5 (0.7-2.9)	1.4 (0.7-2.5)	1.4 (0.8-2.5)	1.3 (0.7-2.2)	1.4 (0.8-2.3)	1.3 (0.96-1.7)	1.4 (0.8-2.3)
**N in known imprinted**	9	11	13	13	17	59	201
**N not in known imprinted**	389	387	385	385	381	339	197

Supplementary Table 1. Test for enrichment/depletion of POE-influenced CpG sites in and around known imprinted regions. The results of enrichment tests are presented for all 984 candidate POE-influenced CpGs, the 586 candidate POE-influenced CpGs amongst these with strong POE evidence and the remaining 398 candidate POE-influenced CpGs with moderate POE evidence. The tests for enrichment were carried out using exact location matches for CpG site and known imprinting region location or relaxing this criterion to allow for matches within a range of distances to the known imprinted region (0: exact match, Within 2 kb, Within 5 kb, Within 10 kb, Within 20 kb, Within 250 kb, Within 2 Mb).We show the p value for the enrichment test (P (Fisher exact)), the enrichment odds ratio (Odds Ratio) and the number of POE-influenced CpG sites in or outside known imprinted regions accounting for the relevant distance matching in each category (N in known imprinted, N not in known imprinted

Source	Description
PMID_27843151	alcohol consumption measured as categorical variable
PMID_27843151	alcohol consumption measured as continuous variable
PMC_5357865	all-cause mortality
PMID_25583993	blood lipid
PMID_28002404	BMI
PMID_27955697	chronic low grade inflammation
PMID_29086770	education
PMID_28194238	blood levels of triglycerides, high-density lipoprotein cholesterol (HDL), low-density lipoprotein cholesterol (LDL), and total cholesterol
PMID_27651444	current vs never smoker
PMID_27651444	former vs never smoker
PMID_27651444	smoking as continuous variable (packed years)
PMC_4609112	smoking associated CgGs that were replicated

Supplementary Table 2. List of published EWAS used to annotate. POE-influenced CpG sites. We provide a reference identification (Source) and the trait subject to EWAS for each of the references (Description)

Target CpG	VC pattern	Chromosome	Position (bp)	CpG-SNP distance (bp)	Index CpG	POE		
						P value	Est	SE
**cg02219360**	Complex	11	2721409	16066	cg09518720	1.11×10^{-13}	-0.042	0.006
**cg09518720**	Complex	11	2721591	16248	cg09518720	$<10^{-170}$	-0.166	0.003
**cg14958441**	Complex	11	2721610	16267	cg09518720	8.04×10^{-169}	-0.103	0.003
**cg05884032**	Paternal	18	76740088	*trans*	cg05884032	7.64×10^{-108}	0.180	0.008
**cg23776532**	Paternal	13	53029591	*trans*	cg23776532	1.47×10^{-32}	-0.073	0.006

Supplementary Table 3. CpG sites affected in *cis* and *trans* (Target CpG) by POE-mQTL SNP rs231356 (located in chromosome 11) . The table shows the imprinting pattern inferred from the variance component analyses (VC imprinting), the location of the CpG sites (Chromosome, Position (bp)), the distance between the CpG sites and the SNP (CpG-SNP distance (bp)), the strength of evidence (P value), estimated POE (Est) and its standard error (SE) from the POE-mQTL analysis. Index CpG gives information about correlations amongst CpG sites.

Trait	Abbreviation	Transformation	Description
Alcohol consumption	Alcohol	log	Alcohol consumption
Ankle Brachial Index	ABI	log	Ratio of lower legs blood pressure to arms blood pressure
Average diastolic blood pressure	DBP	log	Average diastolic blood pressure
Average heart rate	HR	log	Average heart rate
Average systolic blood pressure	SBP	log	Average systolic blood pressure
Blood urea nitrogen	BUN	log	Blood urea nitrogen levels
Body Fat	%Fat	NA	Percentage of body fat (bioimpedance)
Body mass index	BMI	log	Weight/Height^2
Chronic pain grade questionnaire	CP	NA	Questionnaire to evaluate overall severity of chronic pain
Creatinine level	CREAT	log	Creatinine levels
Dairy intake	DAIRY	NA	Weekly dairy intake
Depression (Self-declared)	DEP_SD	NA	Self-declared depression
Education years	EDU	NA	Years of education
Egg intake	EGGS	NA	Weekly egg intake
Fish intake	FISH	NA	Weekly fish intake
Fruit intake	FRUIT	NA	Weekly fruit intake
General cognitive ability	g	NA	General cognitive ability (First unrotated principal component of the four cognitive tests)
General health questionnaire	GHQ	log	General health questionnaire assessed using Likert scale
Glucose	GLUC	log	Glucose level
HDL cholesterol	HDL	log	High-density lipoprotein cholesterol
Height	Height	NA	Height
Hips	Hip	log	Hip circumference
Major depressive disorder	MDD	NA	Diagnosed major depressive disorder
Meat intake	MEAT	NA	Weekly meat intake
Moderate Physical Activity	MOD_PA	log	Hours per week of Moderate Activity
Neuroticism	NEURO	boxcox	Neuroticism
Poultry intake	POULTRY	NA	Poultry frequencies
Smoking	SMK	rank	Smoking (packs per year)
Total cholesterol level	TC	log	Total cholesterol level
Vegetable intake	VEG	NA	Weekly vegetable intake
Vigorous Physical Activity	VIG_PA	log	Hours per week of Vigorous Activity
Waist circumference	WC	log	Waist circumference
Waist-to-hip ratio	WHR	log	Waist/Hips
Weight	Weight	log	Weight of participant

Supplementary Table 4. Brief description (Description) of the phenotypes used in the study, with names (Trait) and abbreviations used in the paper text and tables (Abbreviation), and transformations performed prior to analysis (Transformation)

All candidate CpGs (N=984)						
	**Observed_paternal**	**Expected_paternal**	**Observed_maternal**	**Expected_maternal**	**Observed_complex**	**Expected_complex**
**CpG-trait association sig**	23	7.55	5	10.51	19	28.95
**CpG-trait association not sig**	135	150.45	215	209.49	587	577.05
**Strong POE Evidence (N=586)**						
	**Observed_paternal**	**Expected_paternal**	**Observed_maternal**	**Expected_maternal**	**Observed_complex**	**Expected_complex**
**CpG-trait association sig**	14	3.7	3	6.2	6	13.1
**CpG-trait association not sig**	80	90.3	156	152.8	327	319.9
**Moderate POE Evidence (N=398)**						
	**Observed_paternal**	**Expected_paternal**	**Observed_maternal**	**Expected_maternal**	**Observed_complex**	**Expected_complex**
**CpG-trait association sig**	9	3.9	2	3.7	13	16.5
**CpG-trait association not sig**	55	60.1	59	57.3	260	256.5

Supplementary Table 5. Observed and expected imprinting patterns at POE-influenced CpG sites with or without significant (at per-trait level) methylation level vs. trait associations. We considered all 984 candidate POE-influenced CpGs, the 586 candidate POE-influenced CpGs amongst these with strong POE evidence and the remaining 398 candidate POE-influenced CpGs with moderate POE evidence (All candidate CpGs (N=984), Strong POE Evidence (N=586), Moderate POE Evidence (N=398)). For each of the three sets of CpG sites (all: 984, strong POE evidence: 586 and moderate POE evidence: 398), we estimated the expected distribution of POEs (complex, paternal, maternal; Expected_complex, Expected_paternal, Expected_maternal) for both the group of POE-influenced CpG sites with and without significant trait associations (CpG-trait association sig, CpG-trait association not sig), and compared these distributions to the observed ones (Observed_complex, Observed_paternal, Observed_maternal).

Trait	u_pvalue	bonf_u_pvalue
HDL	3.77E-14	1.28E-12
VEG	1.33E-07	4.52E-06
Weight	2.52E-07	8.57E-06
BMI	3.63E-07	1.24E-05
%Fat	2.40E-06	8.17E-05
WC	4.84E-06	1.65E-04
g	1.93E-04	6.57E-03
UREA	0.00	0.07
ABI	0.80	1.00
DBP	0.73	1.00
HR	1.00	1.00
SBP	0.10	1.00
CP	0.17	1.00
CREAT	0.22	1.00
DAIRY	1.00	1.00
DEP_SD	0.79	1.00
EGGS	1.00	1.00
Eysenk	1.00	1.00
FISH	0.86	1.00
FRUIT	0.04	1.00
GLUC	0.16	1.00
Height	0.41	1.00
Hip	0.22	1.00
GHQ	1.00	1.00
MDD	0.87	1.00
MEAT	0.90	1.00
MOD_PA	0.10	1.00
SMK	1.00	1.00
POULTRY	1.00	1.00
ALCOHOL	0.13	1.00
TC	0.60	1.00
VIG_PA	0.05	1.00
WHR	0.49	1.00
EDU	0.07	1.00

Supplementary Table 6. Mann-Whitney U test results for test statistics (-log(P)) of association test between methylation levels at POE-influenced CpG sites and traits for CpG sites with a detected POE-mQTL association (with strong POE evidence).

Trait	u_pvalue	bonf_u_pvalue
HDL	1.97E-16	6.70E-15
VEG	2.99E-08	1.02E-06
g	3.28E-07	1.11E-05
CREAT	3.64E-07	1.24E-05
BMI	4.82E-07	1.64E-05
Weight	5.63E-06	1.91E-04
EDU	1.26E-04	4.29E-03
ALCOHOL	2.20E-04	7.47E-03
%Fat	6.69E-04	2.28E-02
SBP	1.44E-03	4.90E-02
WC	0.00	0.08
FRUIT	0.02	0.55
VIG_PA	0.86	1.00
POULTRY	1.00	1.00
UREA	0.24	1.00
DBP	0.15	1.00
Hip	0.24	1.00
WHR	0.31	1.00
HR	1.00	1.00
FISH	0.79	1.00
DEP_SD	1.00	1.00
GLUC	1.00	1.00
Height	0.11	1.00
DAIRY	1.00	1.00
MDD	0.86	1.00
CP	0.58	1.00
EGG	1.00	1.00
SMK	1.00	1.00
TC	0.97	1.00
MEAT	0.68	1.00
MOD_PA	0.68	1.00
GHQ	0.93	1.00
API	1.00	1.00
Eysenk	1.00	1.00

Supplementary Table 7. Mann-Whitney U test results for test statistics (-log(P)) of association test between methylation levels at POE-influenced CpG sites and traits for CpG sites without a detected POE-mQTL association (with moderate POE evidence). The trait name (Trait), the test p-value (u_pvalue) and the p-value adjusted for multiple testing (bonf_u_pvalue) are presented.

Tested effect	N	Effect size	SE	P value	Additional description
**SEX x POE**	6980	0.0148	0.0046	1.31E-03	
**Age (10th bin, age>47) x POE**[1]	6980	-0.0194	0.0099	4.91E-02	Based on the 10 deciles of age, the sample was divided into 10 bins, from youngest to oldest (see age ranges below) and we tested the interaction between POE and the age-bins (as categorical variable). A significant POE was detected in the 10th bin, over and above the population POE estimate.

Supplementary Table 8. POE by sex interaction effect (SEX x POE) and POE by age interaction effect (using age bins[1]) on waist circumference at SNP rs6100212 (*cis*-POE-mQTL) in GS:SFHS. For each interaction effect (Tested effect: SEX x POE, Age (10th bin, age>47) x POE), table columns show the sample size used in the analysis (N), de size of the interaction effect (Effect size; for the POE by age interaction, we present the effect estimate of the POE for the 10th bin), its standard error (SE) and an indication of its significance (P value), as well as some additional information (Additional description).

[1]For the analysis of POE by age interaction, we divided the sample into 10 bins with following age ranges and sample sizes: bin1: x <=21,N=921; bin2: 21< x <=23,N=521; bin3: 23< x <=26,N=690; bin4: 26< x <=29,N=684; bin5: 29< x <=33, N=882; bin6: 33< x <=36, N=707; bin7: 36< x <=39,N=701; bin8: 39< x <=42, N=593; bin9: 42< x <=47, N=641; bin10: x>47, N=641. The POE interaction term estimate was largest for the 10th bin, and we present the effect estimate for that bin in this table.

**Tested effect**	**Age**	**Sex**	**N**	**Ref**	**Alt**	**Effect size**	**SE**	**T score**	**P value**
POE	All	All	6980	C	G	-0.013	0.002	-5.17	2.34E-07
POE	All	Female	4025	C	G	-0.019	0.003	-5.43	5.69E-08
POE	All	Male	2955	C	G	-0.005	0.003	-1.48	1.38E-01
POE	bin1: x <=21	All	921	C	G	-0.010	0.006	-1.65	9.87E-02
POE	bin2: 21< x <=23	All	521	C	G	-0.003	0.009	-0.31	7.58E-01
POE	bin3: 23< x <=26	All	690	C	G	-0.008	0.008	-0.98	3.28E-01
POE	bin4: 26< x <=29	All	684	C	G	-0.015	0.008	-1.79	7.31E-02
POE	bin5: 29< x <=33	All	882	C	G	-0.015	0.008	-1.83	6.65E-02
POE	bin6: 33< x <=36	All	707	C	G	-0.010	0.008	-1.34	1.79E-01
POE	bin7: 36< x <=39	All	701	C	G	-0.020	0.008	-2.58	9.95E-03
POE	bin8: 39< x <=42	All	593	C	G	-0.001	0.008	-0.18	8.57E-01
POE	bin9: 42< x <=47	All	641	C	G	-0.013	0.008	-1.62	1.05E-01
POE	bin10: x>47	All	641	C	G	-0.024	0.007	-3.27	1.09E-03
POE	x > 47	Female	396	C	G	-0.030	0.010	-2.97	2.93E-03
POE	x > 47	Male	245	C	G	-0.012	0.010	-1.25	2.12E-01
POE	x <= 47	Female	3729	C	G	-0.017	0.004	-4.66	3.09E-06
POE	x <= 47	Male	2710	C	G	-0.004	0.003	-1.15	2.52E-01

Supplementary Table 9. POE effects at SNP rs6100212 (cis-POE-mQTL) on waist circumference in different age/sex groups in GS:SFHS. We estimated the POE at rs6100212 using all the sample and males and females separately. Similarly, we estimated POE on waist circumference separately for different age ranges (see caption for table S16). In an additional analysis we split the population into males and females over and under the age of 47. Tested effect (POE) indicates that we test the POE, and further columns on the table indicate which age and sex groups are considered in each analysis (Age: All, bin1: x <=21, bin2: 21< x <=23, bin3: 23< x <=26, bin4: 26< x <=29, bin5: 29< x <=33, bin6: 33< x <=36, bin7: 36< x <=39, bin8: 39< x <=42, bin9: 42< x <=47, bin10: x>47, x > 47, x <= 47; Sex: All, Female, Male), the sample size used (N), the reference and alternative alleles at the SNP (Ref, Alt) and the POE size estimate (Effect size), its standard error (SE), the test statistic (T score) and the p-value (P value). Red text indicates the subgroup of individuals with the largest point estimate of the POE effect.

Tested effect	Age	Sex	N	Ref	Alt	Effect size	SE	T score	P value
POE	All	All	4249	C	G	-0.001	0.003	-0.46	6.45E-01
POE	All	Female	2579	C	G	-0.003	0.004	-0.64	5.22E-01
POE	All	Male	1670	C	G	0.000	0.004	0.08	9.36E-01
POE	x > 47	All	198	C	G	-0.034	0.022	-1.53	1.31E-01
POE	x <= 47	All	4051	C	G	-0.001	0.003	-0.35	7.24E-01
POE	x > 47	Female	130	C	G	-0.094	0.063	-1.50	1.51E-01
POE	x > 47	Male	68	C	G	Not enough degrees of freedom to perform the test			
POE	x <= 47	Female	2449	C	G	-0.001	0.004	-0.32	7.49E-01
POE	x <= 47	Male	1602	C	G	-0.001	0.004	-0.15	8.78E-01

Supplementary Table 10. POE effects at SNP rs6100212 (*cis*-POE-mQTL) on waist circumference in different age/sex groups in UK-Biobank. Following GS:SFHS analyses, we estimated the POE at rs6100212 using all the sample and males and females separately. Similarly, we estimated POE on waist circumference separately for different age ranges (over and under the age of 47). In an additional analysis we split the population into males and females over and under the age of 47. Tested effect (POE) indicates that we test the POE, and further columns on the table indicate which age and sex groups are considered in each analysis (Age: All, x > 47, x <= 47; Sex: All, Female, Male), the sample size used (N), the reference and alternative alleles at the SNP (Ref, Alt) and the POE size estimate (Effect size), its standard error (SE), the test statistic (T score) and the p-value (P value). Red text indicates the subgroup of individuals with the largest point estimate of the POE effect.

Supplementary References

1. Delaneau, O., J. Marchini, and J.F. Zagury, *A linear complexity phasing method for thousands of genomes.* Nat Methods, 2012. **9**(2): p. 179-81.
2. McCarthy, S., et al., *A reference panel of 64,976 haplotypes for genotype imputation.* Nat Genet, 2016. **48**(10): p. 1279-83.
3. Fortin, J.P., E. Fertig, and K. Hansen, *shinyMethyl: interactive quality control of Illumina 450k DNA methylation arrays in R.* F1000Res, 2014. **3**: p. 175.
4. Amador, C., et al., *Recent genomic heritage in Scotland.* Bmc Genomics, 2015. **16**(1): p. 437.
5. Pidsley, R., et al., *A data-driven approach to preprocessing Illumina 450K methylation array data.* BMC Genomics, 2013. **14**: p. 293.
6. Zhou, W.D., P.W. Laird, and H. Shen, *Comprehensive characterization, annotation and innovative use of Infinium DNA methylation BeadChip probes.* Nucleic Acids Research, 2017. **45**(4).
7. McCartney, D.L., et al., *Identification of polymorphic and off-target probe binding sites on the Illumina Infinium MethylationEPIC BeadChip.* Genomics Data, 2016. **9**: p. 22-24.
8. Fortin, J.P., T.J. Triche, and K.D. Hansen, *Preprocessing, normalization and integration of the Illumina HumanMethylationEPIC array with minfi.* Bioinformatics, 2017. **33**(4): p. 558-560.
9. Du, P., et al., *Comparison of Beta-value and M-value methods for quantifying methylation levels by microarray analysis.* Bmc Bioinformatics, 2010. **11**.
10. Lehne, B., et al., *A coherent approach for analysis of the Illumina HumanMethylation450 BeadChip improves data quality and performance in epigenome-wide association studies (vol 16, 37, 2015).* Genome Biology, 2016. **17**.
11. Robins, C., et al., *Testing Two Evolutionary Theories of Human Aging with DNA Methylation Data.* Genetics, 2017. **207**(4): p. 1547-1560.
12. Yang, J., et al., *GCTA: a tool for genome-wide complex trait analysis.* Am J Hum Genet, 2011. **88**(1): p. 76-82.
13. Zaitlen, N., et al., *Using Extended Genealogy to Estimate Components of Heritability for 23 Quantitative and Dichotomous Traits.* Plos Genetics, 2013. **9**(5): p. e1003520.
14. Xia, C., et al., *Pedigree- and SNP-Associated Genetics and Recent Environment are the Major Contributors to Anthropometric and Cardiometabolic Trait Variation.* PLoS Genet, 2016. **12**(2): p. e1005804.
15. Cuellar Partida, G., et al., *Genome-wide survey of parent-of-origin effects on DNA methylation identifies candidate imprinted loci in humans.* Hum Mol Genet, 2018.
16. Lynch, M. and B. Walsh, *Genetics and analysis of quantitative traits*. Vol. 1. 1998: Sinauer Sunderland, MA.
17. Lawson, H.A., J.M. Cheverud, and J.B. Wolf, *Genomic imprinting and parent-of-origin effects on complex traits.* Nat Rev Genet, 2013. **14**(9): p. 609-17.
18. Bycroft, C., et al., *The UK Biobank resource with deep phenotyping and genomic data.* Nature, 2018. **562**(7726): p. 203.
19. Manichaikul, A., et al., *Robust relationship inference in genome-wide association studies.* Bioinformatics, 2010. **26**(22): p. 2867-2873.
