# Supplementary material for: Parent of origin genetic effects on methylation in humans are common and influence complex trait variation
Source: Nat Commun. 2019 Mar 27;10:1383. doi: 10.1038/s41467-019-09301-y (PMC6437195; doi:10.1038/s41467-019-09301-y)
Supplement: Supplementary file 3 — Description of Additional Supplementary Files [file 41467_2019_9301_MOESM3_ESM.pdf]

Description of Additional Supplementary Files

Supplementary Data 1. Genomic description of the 984 POE-influenced CpG sites. The table displays the name of each of the CpG sites (CpG) with its physical location in the genome (chromosome: CHR and base pair position: Position), nearest gene (Gene) and location relative to it (Genic: upstream;downstream, intronic, exonic, intergenic, downstream, UTR5, upstream, ncRNA_intronic, UTR3, ncRNA_exonic) and location relative to known imprinted regions (Novel (> 2 Mb of known region): the CpG was not previously detected as a POE influenced CpG, Replication (< 2kb of known region): the CpG is located within 2KB distance of a known imprinted region)). The POE pattern inferred as most likely for the POE-influenced CpG by the POE-targeted variance component analysis (VC Selected Model: Complex, Paternal, Maternal) is shown, together with the p-value from the log-likelihood ratio test for the POE component in the complex, paternal and maternal POE models, and the selected model (amongst the afore mentioned ones) (P (LRT POEcomplex), P (LRT POEpaternal), P (LRT POEmaternal, P (LRT POE SELECTED)). Since there are correlations between methylation levels at CpG sites and we have created a list of independent (Index) POE-influenced CpG sites (see main text **Assessment of number of independent methylation CpGs**), we give information on the index status of each of the POE-influenced CpGs (Index cpg (1=Yes,0=No); 1=Yes: the CpG is one of the 733 independent index CpGs). The strength of evidence for POE as rated based on Table 3 criteria is given in Strength of Evidence (Strong, Moderate). An indication on the test for POE at a specific CpG site passing a conservative Bonferroni corrected threshold is given in the column Passes Bonferroni.

Supplementary Data 2. Functional region based enrichment/depletion results for the 984 POE-influenced CpGs. This table shows the results of functional region based enrichment/depletion analyses for the 984 POE-influenced CpGs, across 6 categories of annotations (Category: Chromatin Status, EWAS hits, genic substructure, GWAS hits, Histone (modifications) and TFBS (transcription factor binding sites)). Chromatin status, Histone and TFBS information has been gathered from cell lines (Cell.line) GM12878 and K562. For each annotation (Annotation; full name and description in Full Annotation Name, Description), the odds ratio for enrichment/depletion (OR) is presented together with the higher and lower bound of its 95% confidence interval (Fisher OR 95CI (lower), Fisher OR 95CI (higher)), and

indication of the significance of the enrichment/depletion (P value from Fisher exact test and Significance (Yes/No)). To further assist with the interpretation on the table, annotations for which the POE-influenced CpGs are significantly depleted are marked with blue font and annotations for which the POE-influenced CpGs are significantly enriched are marked with red font.

Supplementary Data 3. Gene-set based enrichment test for the 984 POE-influenced CpGs. After assigning CpG sites to their nearest gene if they were located between 5 kb in 5′ and 1 kb in 3′ of the boundary of the gene, we performed a gene-set (pathway) analysis, comparing the gene set we obtain from assigning POE-influenced CpG sites to genes to gene sets (Term) in publicly available databases (Category: GAD_DISEASE_CLASS, GAD_DISEASE, UP_SEQ_FEATURE, KEGG_PATHWAY, UP_KEYWORDS, SMART, INTERPRO, GOTERM_MF_DIRECT, GOTERM_CC_DIRECT, COG_ONTOLOGY, BIOCARTA, GOTERM_BP_DIRECT,REACTOME_PATHWAY,OMIM_DISEASE,BIOGRID_INTERACTION). Fold Enrichment provides a measure of the magnitude of enrichment. To examine the significance of gene–term (gene-set) enrichment we use both a modified Fisher's exact test ((EASE score) in DAVID[1, 2]) and provide its p value (P value) and a multiple testing corrected p value (Benjamini) to control family-wide false discovery rate. Rows in red show gene-sets with a Benjamini p value ≤ 0.05.

Supplementary Data 4. List of significant Cis-POE-mQTLs. To identify the imprinted genetic variants that potentially underlie the observed POEs affecting methylation levels at the 984 candidate CpGs identified by the variance components analyses, a POE-mQTL analysis was performed for each of the 984 CpGs. *Cis*-POE-mQTLs are defined as SNPs showing a significant POE for a methylation CpG and within less than 1 Mb from that CpG. The significance threshold for association was determined via a permutation-based multiple testing correction at the FDR≤0.05 level and was $3.6x10^{-4}$ for *cis*-POE-mQTLs. The table shows each *cis*-POE-mQTL rsid, its chromosome and physical location in base pairs and its alleles (Target SNP, Chromosome (target SNP), Position (target SNP), Ref Allele (SNP), Alt Allele (SNP)), as well as its associated CpG site –with chromosome and physical location- (Target CpG, Chromosome (target CpG), Position (target CpG)), its distance to it (Distance target SNP to target CpG (bp)), and the estimated effect size of the POE (Est (POE)) with its associated

standard error (SE (POE)) and p value (p-value (POE)). For each *cis*-POE-mQTL-CpG site pair, we provide information on the independent (and most significant) CpG site and *cis*-POE-mQTL they are represented by (Index CpG, Index SNP; see main text **Assessment of number of independent methylation CpGs** and **POE-mQTL: Parent-of-origin effect methylation quantitative trait loci analyses**).

Supplementary Data 5. List of significant Trans-POE-mQTLs. To identify the imprinted genetic variants that potentially underlie the observed POEs affecting methylation levels at the 984 candidate CpGs identified by the variance components analyses, a POE-mQTL analysis was performed for each of the 984 CpGs. *Trans*-POE-mQTLs are defined as SNPs showing a significant POE for a methylation CpG located more than 5 Mb away from that CpG. The significance threshold for association was determined via a permutation-based multiple testing correction at the FDR≤0.05 level and was 2.19×10^{-9} for *trans*-POE-mQTLs. The table shows each *trans*-POE-mQTL rsid, its chromosome and physical location in base pairs and its alleles (Target SNP, Chromosome (target SNP), Position (target SNP), Ref Allele (SNP), Alt Allele (SNP)), as well as its associated CpG site –with chromosome and physical location- (Target CpG, Chromosome (target CpG), Position (target CpG)) and the estimated effect size of the POE (Est (POE)) with its associated standard error (SE (POE)) and p value (p-value (POE)). For each *trans*-POE-mQTL-CpG site pair, we provide information on the independent (and most significant) CpG site and *trans*-POE-mQTL they are represented by (Index CpG, Index SNP; see main text **Assessment of number of independent methylation CpGs** and **POE-mQTL: Parent-of-origin effect methylation quantitative trait loci analyses**).

Supplementary Data 6. Variance component analysis results for POEs in 260 CpGs detected as POE-influenced in ALSPAC[3]. 260 CpGs of the 327 associated with POE from 199 genetic variants in ALSPAC, were also analysed in our study. For these, we present name of each of the CpG sites (CpG) with its physical location in the genome (chromosome: Chromosome and base pair position: Position), nearest gene (Gene) and location relative to it (Genic: upstream;downstream, intronic, exonic, intergenic, downstream, UTR5, upstream, ncRNA_intronic, UTR3, ncRNA_exonic) and indication of replication of the ALSPAC finding in GS:SFHS (Replicated in GS:SFHS: Yes/No). The p-value from the log-likelihood ratio test for the POE component in the complex, paternal and maternal POE models, and the selected

model (amongst the afore mentioned ones, GS:SFHS: VC Selected model) in GS:SFHS is shown (GS:SFHS: P (LRT POE Complex), GS:SFHS: P (LRT POE Paternal), GS:SFHS: P (LRT POE Maternal), GS:SFHS: P (LRT POE Selected)) as are the p-values of the ALSPAC analyses at different developmental stages (ALSPAC: Birth P, ALSPAC: Child P, ALSPAC: Adolescence P).

Supplementary Data 7. Functional region based enrichment/depletion results for 586/984 CpG sites with at least an associated POE-mQTL. This table shows the results of functional region based enrichment/depletion analyses for the 586 CpG sites with an associated POE-mQTL, across 6 categories of annotations (Category: Known imprinted, Chromatin Status, EWAS hits, genic substructure, Histone (modifications) and TFBS (transcription factor binding sites)). Chromatin status, Histone and TFBS information has been gathered from cell lines (Cell.line) GM12878 and K562. For each annotation (Annotation; full name and description in Full annotation name, Description), the odds ratio for enrichment/depletion (OR) is presented together with the higher and lower bound of its 95% confidence interval (Fisher OR 95CI (lower), Fisher OR 95CI (higher)), and indication of the significance of the enrichment/depletion (P value from the Fisher exact test and Significance (Yes/No)). To further assist with the interpretation on the table, annotations for which the CpG sites with an associated POE-mQTL are significantly depleted are marked with blue font and annotations for which the CpG sites with an associated POE-mQTL are significantly enriched are marked with red font.

Supplementary Data 8. Functional region based enrichment/depletion results for 398/984 CpG sites without an associated POE-mQTL. This table shows the results of functional region based enrichment/depletion analyses for the 398 CpG sites with no associated POE-mQTL, across 6 categories of annotations (Category: Known imprinted, Chromatin Status, EWAS hits, genic substructure, Histone (modifications) and TFBS (transcription factor binding sites)). Chromatin status, Histone and TFBS information has been gathered from cell lines (Cell.line) GM12878 and K562. For each annotation (Annotation; full name and description in Full annotation name, Description), the odds ratio for enrichment/depletion (OR) is presented together with the higher and lower bound of its 95% confidence interval (Fisher OR 95CI (lower), Fisher OR 95CI (higher)), and indication of the significance of the

enrichment/depletion (P value from the Fisher exact test and Significance (Yes/No)). To further assist with the interpretation on the table, annotations for which the CpG sites with no associated POE-mQTL are significantly depleted are marked with blue font and annotations for which the CpG sites with no associated POE-mQTL are significantly enriched are marked with red font.

Supplementary Data 9. Significant associations between POE-influenced CpGs and traits. We list 81 methylation-trait associations between 47 CpG sites and 17 traits at per-trait significance level in at least one of the groups of CpG sites with evidence of POEs (CpG group with strong evidence and CpG group with moderate evidence of POEs). The table displays the name of each of the CpG sites (CpG) with its physical location in the genome (chromosome: Chromosome and base pair position: Position), nearest gene (Gene) and location relative to it (Genic Region: upstream;downstream, intronic, exonic, intergenic, downstream, UTR5, upstream, ncRNA_intronic, UTR3, ncRNA_exonic). The strength of evidence for POE as rated based on Table 3 criteria is given in Strength of Evidence (Strong, Moderate). The trait affected by variation in methylation levels at each given CpG site is shown (Associated trait), as well as the effect on the trait (Estimate, with standard error (SE)), and the association p-value (P). The POE pattern inferred as most likely for the POE-influenced CpG by the POE-targeted variance component analysis (VC Selected Model: Complex, Paternal, Maternal) is also shown.

Supplementary Data 10. Published trait associations for SNP rs6100212. This *cis*-POE-mQTL causes complex imprinting patterns at 12 DNA methylation sites, waist circumference, BMI, body fat and waist-to-hip ratio. Previous published GWAS[4], that used an additive model in the association analysis and failed to uncover SNP-trait associations for rs6100212 (SNP, with location Pos (hg19)). We present results of this additive association analysis, including information on the study and references (Study, PMID). For each phenotype (Trait; multiple instances of the same phenotype name are possible as we use multiple publications), an association p-value for the additive model from the t test is shown (P value (unadjusted), not adjusted for multiple testing), the effect direction (Direction -/+) and the study sample size (N, N Cases, N Controls, with N= N Controls and N Cases= 0 for population studies).
